# Antibodies from malaria-exposed pregnant women recognize trypsin resistant epitopes on the surface of *Plasmodium falciparum*-infected erythrocytes selected for adhesion to chondroitin sulphate A

**DOI:** 10.1186/1475-2875-3-31

**Published:** 2004-09-06

**Authors:** Lisa Sharling, Anders Enevold, Kordai MP Sowa, Trine Staalsoe, David E Arnot

**Affiliations:** 1 Institute of Cell, Animal and Population Biology, University of Edinburgh, King's Buildings, West Mains Road, Edinburgh, EH9 3JT, Scotland, UK; 2 Centre for Medical Parasitology, Department of Infectious Diseases M7641. Rigshospitalet, Blegdamsvej 9, 2100 Copenhagen Ø, Denmark

## Abstract

**Background:**

The ability of *Plasmodium falciparum*-infected erythrocytes to adhere to the microvasculature endothelium is thought to play a causal role in malaria pathogenesis. Cytoadhesion to endothelial receptors is generally found to be highly sensitive to trypsinization of the infected erythrocyte surface. However, several studies have found that parasite adhesion to placental receptors can be markedly less sensitive to trypsin. This study investigates whether chondroitin sulphate A (CSA) binding parasites express trypsin-resistant variant surface antigens (VSA) that bind female-specific antibodies induced as a result of pregnancy associated malaria (PAM).

**Methods:**

Fluorescence activated cell sorting (FACS) was used to measure the levels of adult Scottish and Ghanaian male, and Ghanaian pregnant female plasma immunoglobulin G (IgG) that bind to the surface of infected erythrocytes. *P. falciparum *clone FCR3 cultures were used to assay surface IgG binding before and after selection of the parasite for adhesion to CSA. The effect of proteolytic digestion of parasite erythrocyte surface antigens on surface IgG binding and adhesion to CSA and hyaluronic acid (HA) was also studied.

**Results:**

*P. falciparum *infected erythrocytes selected for adhesion to CSA were found to express trypsin-resistant VSA that are the target of naturally acquired antibodies from pregnant women living in a malaria endemic region of Ghana. However *in vitro *adhesion to CSA and HA was relatively trypsin sensitive. An improved labelling technique for the detection of VSA expressed by CSA binding isolates has also been described.

**Conclusion:**

The VSA expressed by CSA binding *P. falciparum *isolates are currently considered potential targets for a vaccine against PAM. This study identifies discordance between the trypsin sensitivity of CSA binding and surface recognition of CSA selected parasites by serum IgG from malaria exposed pregnant women. Thus, the complete molecular definition of an antigenic *P. falciparum *erythrocyte surface protein that can be used as a malaria in pregnancy vaccine has not yet been achieved.

## Background

Rapid clearance of parasitaemia following transfusion of IgG from malaria immune adults to clinically ill recipients illustrates that naturally acquired antibodies have a parasite clearing role in human malaria infection [1-3]. Neither the nature of the protective immune response nor the target antigens and epitopes recognized by infection clearing antibodies are fully understood. Evidence is accumulating to suggest that the acquisition of antibodies binding the VSA on infected erythrocytes plays a major role in the development of age and exposure dependent immunity [4-8]. The evidence for protective anti-VSA responses is particularly strong for the PAM syndrome [9,10].

PAM is characterized by the sequestration of *Plasmodium falciparum *infected erythrocytes in the intervillous spaces of the placenta. Infected erythrocytes adhere to low-sulphated forms of CSA present on the extracellular proteoglycan matrix of syncytiotrophoblasts [11]. *In vitro *selection of infected erythrocytes for adhesion to CSA concomitantly selects for expression of VSA that share characteristics with postnatal placental isolates. Thus plasma antibodies from malaria exposed pregnant, or multi-gravid women, recognize the VSA of CSA binding parasites (here referred to as VSA_PAM_). These sera can also block adhesion of CSA-selected infected erythrocytes to CSA *in vitro *[12]. Interestingly, antibodies that bind CSA-selected parasites and block adhesion are not acquired by malaria-exposed males. There is a striking female-specific antibody response recognizing both *in vitro *CSA-selected parasites [12,13] and *P. falciparum *isolates taken from infected placentae at delivery [14-16]. Furthermore, the levels of CSA-adhesion blocking plasma IgG have been shown to increase with adult female parity. Recent immuno-epidemiological studies also show a strong positive correlation between the levels of antibodies that recognize the infected erythrocyte surface[15], the level of CSA-adhesion blocking antibody [17] and positive birth outcomes as measured by birth weight.

PAM is, thus, the clearest example in malaria pathology research of a strong association between infected erythrocyte sequestration and a particular disease syndrome. The VSA recognized by female-specific, parity-dependent antibodies are, therefore, rational and exceptionally interesting candidates for inclusion in an experimental vaccine to protect women against PAM, a major cause of stillbirth, maternal anaemia and low birthweight.

To date, the best characterized VSA is *P. falciparum *erythrocyte membrane protein 1 (PfEMPl), a polymorphic, high molecular weight membrane protein (200–450 kDa) encoded by the *var *multi-gene family [18-20]. Members of the PfEMP-1 family function as adhesion molecules binding to various host endothelial receptors. They are situated in the knob-like protrusions associated with the parasitized erythrocyte surface.

Since *var *genes encode large extracellular domains rich in lysine and arginine residues, it is not surprising that PfEMP-1 molecules and adhesion to endothelial receptors have been reported to be highly sensitive to trypsin treatment [18,21-24]. Less expected was the finding that parasite adhesion to the placental receptor CSA, when immobilized [25-27] or when cell surface associated [28,29], can be relatively trypsin resistant. This study investigates the protease-sensitivity profile of the VSA_PAM _expressed by CSA-selected parasite clone FCR3 with regard to recognition by antibodies acquired during PAM and adhesion to placental receptors.

## Methods

### Parasite isolates

Parasites were maintained in group O erythrocytes under standard conditions [30], using RPMI 1640 medium containing 25 mM HEPES, supplemented with 20 mM glucose, 2 mM glutamine, 25 μg/ml gentamycin and 10% pooled normal human serum. The pH was adjusted to between 7.2 and 7.4 with 1 M NaOH. Culture flasks at 5% haematocrit were gassed with 96% nitrogen, 3% carbon dioxide and 1% oxygen. The laboratory clone FCR3 originates from peripheral blood collected in the Gambia. FCR3CSA was obtained from the Malaria Research and Reference Reagent Resource Centre (ATCC) [31], and was confirmed, using genetic markers to be identical to the laboratory clone FCR3 kept in the original W.H.O. strain registry collection in Edinburgh (D. Walliker, pers. comm.). CSA binding was maintained by panning late stage infected erythrocytes fortnightly on bovine tracheal CSA (10 μg/ml) (Sigma) immobilized on polystyrene Petri dishes (Falcon), as previously described [26]. Prior to protease treatment and analysis by flow cytometry, cultures were synchronized by sorbitol treatment to obtain cultures enriched for late stage parasites.

### Plasma donors

Serum samples from 20 men living in a malaria endemic region of Ghana were pooled to produce the male serum pool. Serum samples collected at the time of birth from the placentas of 15 women living in a malaria endemic region of Ghana were pooled to produce the pregnant female serum pool. This pool included five primigravidae, nine secundigravidae and one multigravid woman. Serum samples from six Scottish malaria naïve individuals were pooled and used as a control.

### Protease treatment

Protease treatment of infected erythrocytes was carried out as previously described [26]. Briefly, samples containing 3 × 10^6 ^cells from sorbitol treated late stage cultures of 8–10 % parasitaemia were washed twice with phosphate-buffered saline (PBS) and then incubated with the appropriate concentration of trypsin-TPCK (Worthington Biochemicals) or pronase (Boehringer-Mannheim) in a final volume of 1.0 ml in PBS, for 10 minutes at 37°C. The reaction was terminated either by adding soybean trypsin inhibitor (Worthington Biochemicals) to a final concentration of 1 mg/ml or by adding 10% human serum. Cells were washed twice with PBS before further use.

### Analysis of VSA specific antibodies by flow cytometry

Flow cytometry was used to measure the levels of plasma IgG binding to the VSA of late stage parasites essentially following the method previously described by Staalsoe *et al *[13,32]. 3 × 10^6 ^cells form late stage *P. falciparum *cultures of 8–10 % parasitaemia were washed twice with PBS. Cells were incubated sequentially with plasma antibodies diluted 1:20 in PBS, goat anti-human IgG diluted 1:200 in PBS (Dako) and fluorescein isothiocyante (FITC)-conjugated rabbit anti-goat (Dako) diluted 1:25 in PBS. All incubations were in a total volume of 100 μl for 30 minutes at room temperature and were followed by two washes with 1 ml of PBS. Samples were analysed immediately on a FACSCAN apparatus (Becton-Dickinson). FITC fluorescence due to cell surface antibody recognition was determined for 5000–10000 ethidium bromide gated infected erythrocytes.

### Modified labeling procedure for FACS analysis

In order to circumvent the non-specific labeling of the VSA by the tertiary antibody, new reagents have been introduced. The procedure follows the method detailed above with the following modifications. A biotinylated rabbit anti-human IgG antibody (DAKO) was used diluted 1:25 to replace the secondary antibody. In the place of a tertiary antibody, FITC-conjugated streptavidin (DAKO) was used at a 1:2000 dilution. In these experiments the control sera was a pool of malaria naïve Danish volunteer serum.

### Binding assays

Human umbilical cord hyaluronic acid (Sigma) and bovine trachea CSA (Sigma) were used at a concentration of 10 μg/ml in PBS (pH 7.2). 20 μl of each receptor was spotted in triplicate onto 5 cm diameter petri dishes (Falcon). Receptors were adsorbed onto the plastic petri dishes overnight at 4°C. 10 μg/ml BSA in PBS was similarly adsorbed as a negative control. Plates were then blocked by removing the receptor solution and adding 20 μl of 2% BSA in PBS. Following the removal of this blocking solution late stage parasites, suspended in 2 ml of complete RPMI-HEPES medium (8–10% parasitaemia, 5% haematocrit), were added to the petri dish. Parasites were incubated with the immobilized receptor for 60 minutes at 37°C with occasional agitation. Unbound cells were removed by four gentle washes with incomplete RPMI-HEPES medium; bound cells were fixed with 0.5% (v/v) glutaraldehyde in PBS for 10 minutes and Giesma Stained. Bound cells were counted by light microscopy. Protease treatment of intact cells was carried out as described above.

### Statistical analysis

Statistical analyses were performed using Analyses of Variance in Minitab 13.30 (Minitab Inc.), using protease, protease concentration and serum pool as explanatory variables. Statistical models were tested for homogeneity of variance and normality of error distributions. Where possible, maximal models with interactions between these variables were fitted first, after which models were minimized by removing nonsignificant (p > 0.05) terms.

## Results

### Concomitant selection of a trypsin-resistant VSA following parasite selection for CSA adhesion

**Figure 1:**
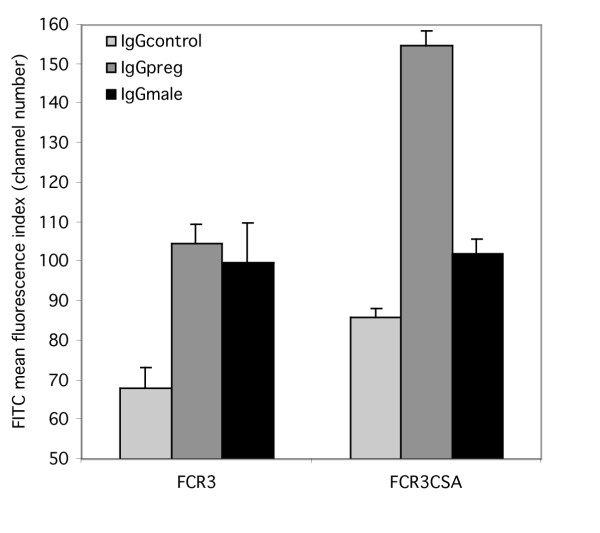
**IgG recognition profiles of parasite clone FCR3 before and after selection for adhesion to CSA. **Following selection of parasite clone FCR3 for adhesion to CSA the expression of variant surface antigens was investigated using FACS. 5000–10000 late stage parasites were gated using ethidium bromide and FITC fluorescence due to serum IgG binding was measured. Serum samples from six Scottish malaria naïve individuals were pooled and used as a control (IgG*control*). Sera from 20 Ghanaian men were pooled to produce the malaria exposed male serum pool (IgG*male*). Sera collected at the time of birth from the placentas of 15 Ghanaian women were pooled to produce the malaria exposed pregnant female serum pool (IgG*preg*). The bar chart shows mean and standard error of the means for five independent experiments.

It was first established that selection of clone FCR3 for adhesion to CSA resulted in the concomitant selection for VSA specifically recognized by plasma IgG from malaria exposed Ghanaian pregnant women (IgG*preg*) (figure 1). However there was no increase in the binding of IgG from a pool of plasma from malaria exposed Ghanaian men (IgG*male*). The unselected FCR3 clone expressed VSA that were equally well recognized by antibodies in the IgG*male *and IgG*preg *serum pools (figure 1). These interactions between serum antibody binding and selection for CSA adhesion were highly significant (F_2,24 _9.5, P = 0.001).

**Figure 2:**
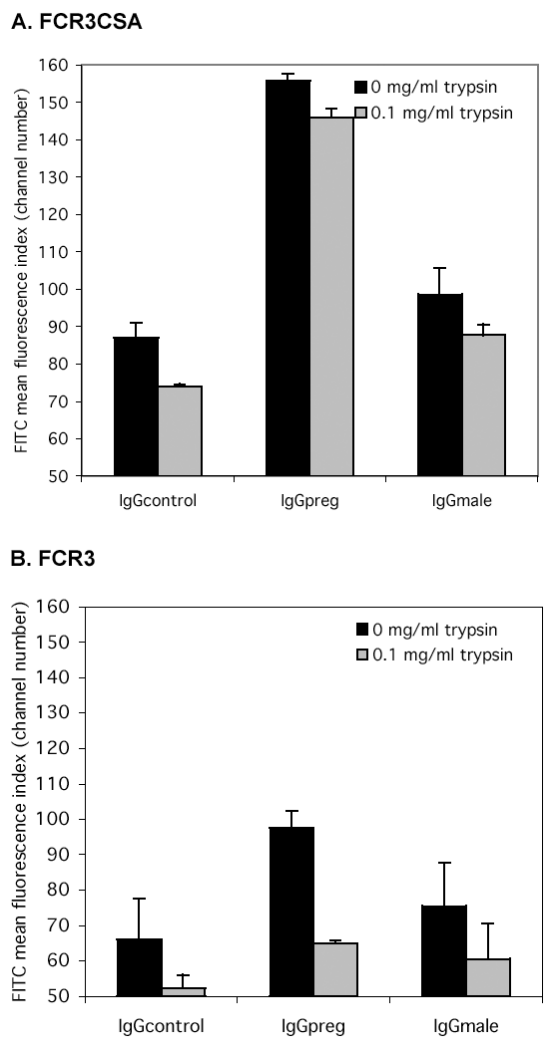
**Serum IgG from malaria exposed pregnant women recognises trypsin-resistant surface epitopes. **Intact infected erythrocytes were treated with 0.1 mg/ml trypsin prior to FACS analysis. Panels A and B show serum IgG binding to the surface of FCR3CSA and FCR3 infected erythrocytes respectively. Serum pools are the same as those described in Figure 1. The bar chart shows mean and standard error of the means for two independent experiments.

The trypsin sensitivity of this VSA/IgG binding interaction and of parasite adhesion to CSA was then measured. Parasitized erythrocyte surface trypsinization at a concentration of 0.1 mg/ml showed that the IgG*preg *binding of FCR3CSA was significantly more trypsin-resistant than was binding of the same serum to the unselected clone (figure 2A &2B; F_1,4_16.4, p = 0.015). Although the mean surface fluorescence due to the IgG*preg *binding of FCR3CSA was slightly reduced by 0.1 mg/ml trypsin this reduction was not significant (figure 2A; F_1,2 _11.3, p = 0.078). The effect of 0.1 mg/ml trypsin on VSA recognition by IgG*male *and IgG*control *was comparable before and after CSA selection of the parasite (figure 2).

**Figure 3:**
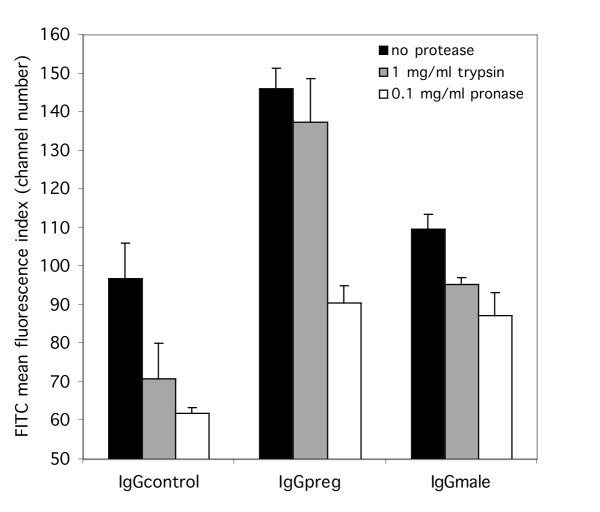
**FCR3CSA expresses surface antigens exhibiting differential protease sensitivity. **Intact infected erythrocytes were treated with 1.0 mg/ml trypsin or 0.1 mg/ml pronase prior to FACS analysis. Serum pools are the same as those described in Figure 1. The bar chart shows mean and standard error of the means for three independent experiments.

The effect of a 10-fold higher trypsin concentration and the effect of the non-specific protease, pronase, on IgG recognition of FCR3CSA was also determined. Trypsinization with 1 mg/ml did not significantly reduce the mean surface fluorescence due to IgG*preg *binding to FCR3CSA (figure 3; F_1,4 _0.35, p = 0.587). However, treatment of the intact infected erythrocyte with 0.1 mg/ml pronase did significantly reduce IgG*preg *recognition of FCR3CSA (figure 3). Pronase treatment also significantly reduced binding of the IgG*male *and IgG*control *serum pools (figure 3; F_4,18_3.1, p = 0.041).

**Figure 4:**
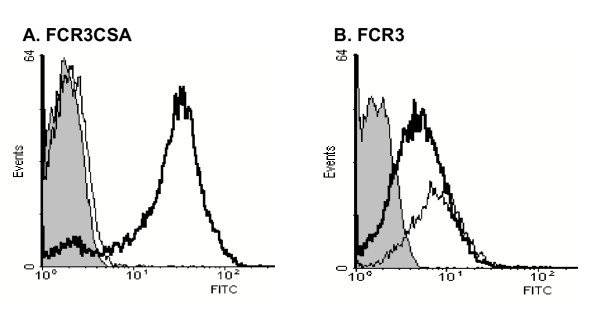
**A modified antibody labelling procedure for FACS analysis of CSA selected parasites. **In order to circumvent the non-specific labelling of FCR3CSA VSA seen when using the FITC rabbit anti-goat tertiary antibody, a biotinylated rabbit anti-human antibody in combination with FITC-conjugated streptavidin was used. Panels A and B show FCR3CSA and FCR3 infected erythrocytes respectively. In these experiments the control serum was a pool of sera from malaria naïve Danish volunteers, here shown as a solid grey histogram. The IgG*male *serum pool is shown as a lightweight line and the IgG*preg *serum pool as a heavyweight line.

Surprisingly, IgG*control *binding to the infected erythrocyte surface increased following CSA selection of the parasite (figure 1); however, this non-immune recognition was found to be significantly more trypsin sensitive than IgGpreg recognition (figure 3; F_4,18_3.11, p = 0.041). This indicates that the epitopes recognized by the IgG*control *serum pool and the epitopes recognized by the IgG*preg *serum pool are distinct entities. An increase in apparent non-immune immunoglobulin binding to the infected erythrocyte surface has been observed for a number of parasite clones after selection for adhesion to CSA (data not shown). The source of this background labelling of FCR3CSA by naïve sera was found to be due to non-specific binding by the FITC-labelled tertiary rabbit anti-goat antibody. By using the modified antibody labelling procedure, which employs a biotin-labelled secondary antibody and FITC-labelled streptavidin, binding of malaria naive IgG to FCR3CSA (mean fluorescence index = 16) was comparable to the unselected parasite (mean fluorescence index = 17). Thus the recognition of VSA_PAM _by malaria naive IgG was abolished (figure 4).

### Discordance between the protease sensitivity of the CSA adhesion interaction and IgG binding

**Figure 5:**
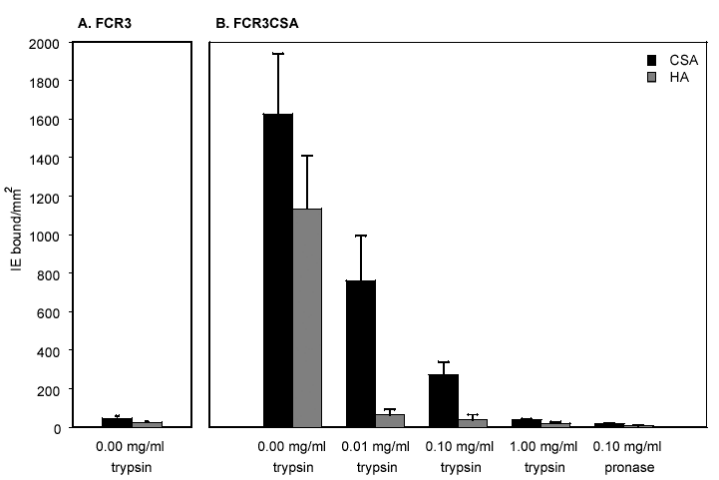
**The effect of increasing concentrations of trypsin on parasite adhesion to immobilised CSA and HA. **Parasite adhesion to 10 μg/ml human umbilical cord HA and bovine trachea CSA, adsorbed onto the plastic petri dishes, was determined following protease treatment of the intact infected erythrocyte. Bound cells were Giemsa stained and counted by light microscopy. Panels A and B show receptor binding for FCR3 and FCR3CSA infected erythrocytes respectively. The bar chart shows mean and standard error of the means for three independent experiments.

Following the identification of trypsin-resistant epitopes that appear to be concomitantly selected with CSA adhesion, the trypsin sensitivity of CSA adhesion itself was determined. FCR3CSA binding to immobilised CSA was markedly more sensitive to trypsin than IgG*preg *recognition of the infected erythrocyte surface (figure 5). Parasite adhesion was reduced by 81% and 91% following treatment with 0.1 mg/ml trypsin and 1 mg/ml trypsin respectively (figure 5). A trypsin concentration of 1 mg/ml reduced binding as efficiently as 0.1 mg/ml pronase, and although 0.1 mg/ml pronase significantly reduced cell surface fluorescence due to IgG*preg *antibody binding, 1 mg/ml trypsin had no significant effect on IgG*preg *antibody binding. There is, thus, significant discordance between the high trypsin sensitivity of CSA adhesion and the relatively trypsin-insensitive binding of IgG*preg *serum antibodies to the infected erythrocyte surface (F_1,8 _14.4, p = 0.005).

Human umbilical cord hyaluronic acid (HA) was also included in these assays to investigate the binding capacity of the CSA selected clone with respect to this receptor. FCR3CSA was found to bind both HA and CSA, although binding to HA was significantly lower (figure 5B; F_3,19 _20.44, p < 0.001), at 71% that observed for CSA. Interestingly, as has previously been shown for other *P. falciparum *isolates [27], the trypsin-sensitivity of parasite adhesion to HA and CSA differed at low trypsin concentrations (0.01 mg/ml) (figure 5B; F_1,8 _7.7, p = 0.024). Parasite adhesion to hyaluronic acid was found to be more sensitive to trypsinization than adhesion to CSA.

## Discussion

The acquisition of antibodies to the surface of placental isolates correlates with protection from malaria in pregnancy and the targets of these antibodies are potential vaccine candidates [13,15]. Two variants of the well characterized VSA, PfEMPl, have been shown to have distinct CSA-binding domains [29,33] and antibodies raised against these domains have been reported to recognize the infected erythrocyte surface [34] and in some cases block parasite adhesion [35,36]. However, in a recent study of *var *gene transcription in CSA-selected clones, a third potential CSA-binding PfEMPl (var2csa) was identified. Var2csa is predicted to possess distinctly different DBL domains and appears to be the major *var *expressed by CSA-selected parasites that are recognized by parity-dependent antibodies [14]. Proteomic analysis of CSA-selected parasites has also identified four additional potential CSA binding PfEMPl molecules [37]. The molecular identity of the surface antigens expressed at the infected erythrocyte surface remains unclear [38]. However, the differential protease sensitivity of the epitopes described here would allow treatment of the infected erythrocyte surface with trypsin thereby simplifying the surface complexity, thus, potentially making proteomic approaches more straightforward.

Although PfEMPl-mediated CSA adhesion appears to play a role in placental malaria the molecular interactions triggering this syndrome are more complex than initially thought. Several studies implicate additional receptors and binding phenotypes of placental parasites, such as non-immune IgM [39], hyaluronic acid [25,27,40] and non-immune IgG [41]. CSA-binding laboratory clones and placental CSA binding isolates also appear to express some parasite encoded surface antigens other than PfEMPl, such as ring surface proteins 1 and 2 (RSP 1 and 2) [42]. Interestingly, a gene 'knock-out' of the CSA binding *var *(FCR3*var*CSA) in parasite clone FCR3 abolishes CSA binding, but the 'knock-out' parasites still bind the syncytio-trophoblast of *ex vivo *placental cryosections [43]. Monoclonal antibodies raised against the CSA binding DBLγ domain also show this domain to be sensitive to surface proteolysis using relatively low trypsin concentrations (100 μg/ml) [34]. It is certainly possible that the trypsin-resistant VSA described here are not of the PfEMPl/CSA binding type.

Surface epitopes of the FCR3CSA parasite are both highly resistant to trypsin and are recognized by antibodies from malaria-exposed pregnant women. This agrees with a number of studies that have found parasite adhesion to placental receptors to be resistant to surprisingly high trypsin concentrations. However, binding assays with the parasite clone used in this study showed CSA and HA adhesion to be relatively trypsin-sensitive. This is also compatible with the results of Beeson and his colleagues who demonstrated trypsin-resistant CSA adhesion to be a clone dependent phenomenon [27]. Another recent study by the same group showed sera that is strongly reactive to the surface of CSA selected parasites is not always capable of inhibiting CSA adhesion [44]. Thus this study supports the view that erythrocyte surface epitopes distinct from those involved in CSA adhesion may be targets of the antibodies acquired during PAM and suggests that these two epitopes could be on different molecules. One further implication for vaccine development is that a candidate vaccine raising only CSA adhesion blocking antibodies may not mimic protective surface reactive gender-specific immune responses.

## Conclusion

This study supports the view that major differences exist between VSA_PAM _and previously characterized VSA. Apart from being recognized only by female sera in a parity-dependent manner, VSA_PAM _show other distinct characteristics such as; i) VSA_PAM _rarely form infected erythrocyte rosettes when compared to CD36 binding VSA [27,45], ii) with the exception of rosetting isolates, non-immune IgM binding is a phenomenon only seen with CSA-binding clones [39], iii) VSA_PAM _do not generally mediate adhesion to CD36 [27,46], and iv) VSA_PAM _mediated adhesion to the placenta and CSA can be resistant to concentrations of trypsin known to remove most PfEMPl molecules from the infected cell surface. In combination with the findings of this study, these distinct properties of VSA_PAM _suggest the involvement of either an unusually protease-resistant PfEMPl structure, such has been shown to exist in the A4tres PfEMPl molecule [47] or an alternative class of VSA in placental adhesion. The differential protease sensitivity exhibited by VSA_PAM _can be exploited in comparative proteomic analysis to aid in the identification of the molecules whose phenotype is described here.

## List of abbreviations

TPCK – L-(tosylamido-2-phenyl) ethyl chloromethyl ketone, CSA – chondroitin sulphate A, PfEMPl – *P. falciparum *erythrocyte protein 1, PAM – Pregnancy associated malaria, VSA – variant surface antigens, VSA_PAM _– variant surface antigens expressed by placental or CSA binding parasites, IgG – immunoglobulin G, DBL-γ-Duffy like binding domain-gamma, FITC – fluorescein isothiocyanate.

## Authors' contributions

LS conceived of the study, maintained *P. falciparum *culture, performed FACS analysis and binding assays, AE performed the modified labelling FACS experiments and participated in manuscript preparation, MS participated in the design of the study, TS helped develop some methodologies used in this study. DA helped conceive and fund the study and write the manuscript. All authors read and approved the final manuscript.

## Declaration

None declared.
